# Multiplex Molecular Point-of-Care Test for Syndromic Infectious Diseases

**DOI:** 10.1007/s13206-021-00004-5

**Published:** 2021-02-15

**Authors:** Hanbi Kim, Hee Jae Huh, Eunkyoung Park, Doo-Ryeon Chung, Minhee Kang

**Affiliations:** 1 Biomedical Engineering Research Center, Smart Healthcare Research Institute, Samsung Medical Center, Seoul, 06351 South Korea; 2 Department of Medical Device Management and Research, SAIHST (Samsung Advanced Institute for Health Sciences & Technology), Sungkyunkwan University, Seoul, 06355 South Korea; 3 Department of Laboratory Medicine and Genetics, Samsung Medical Center, Sungkyunkwan University School of Medicine, Seoul, 06351 South Korea; 4 Center for Infection Prevention and Control, Samsung Medical Center, Seoul, 06351 South Korea; 5 Asia Pacific Foundation for Infectious Diseases (APFID), Seoul, 06367 South Korea; 6 Division of Infectious Diseases, Department of Internal Medicine, Samsung Medical Center, Sungkyunkwan University School of Medicine, Seoul, 06351 South Korea

**Keywords:** Multiplex molecular point-of-care testing, Syndromic infectious disease, Molecular diagnosis, POCT

## Abstract

Point-of-care (POC) molecular diagnostics for clinical microbiology and virology has primarily focused on the detection of a single pathogen. More recently, it has transitioned into a comprehensive syndromic approach that employs multiplex capabilities, including the simultaneous detection of two or more pathogens. Multiplex POC tests provide higher accuracy to for actionable decisionmaking in critical care, which leads to pathogen-specific treatment and standardized usages of antibiotics that help prevent unnecessary processes. In addition, these tests can be simple enough to operate at the primary care level and in remote settings where there is no laboratory infrastructure. This review focuses on state-of-the-art multiplexed molecular point-of-care tests (POCT) for infectious diseases and efforts to overcome their limitations, especially related to inadequate throughput for the identification of syndromic diseases. We also discuss promising and imperative clinical POC approaches, as well as the possible hurdles of their practical applications as front-line diagnostic tests.

## Introduction

**Fig. 1:**
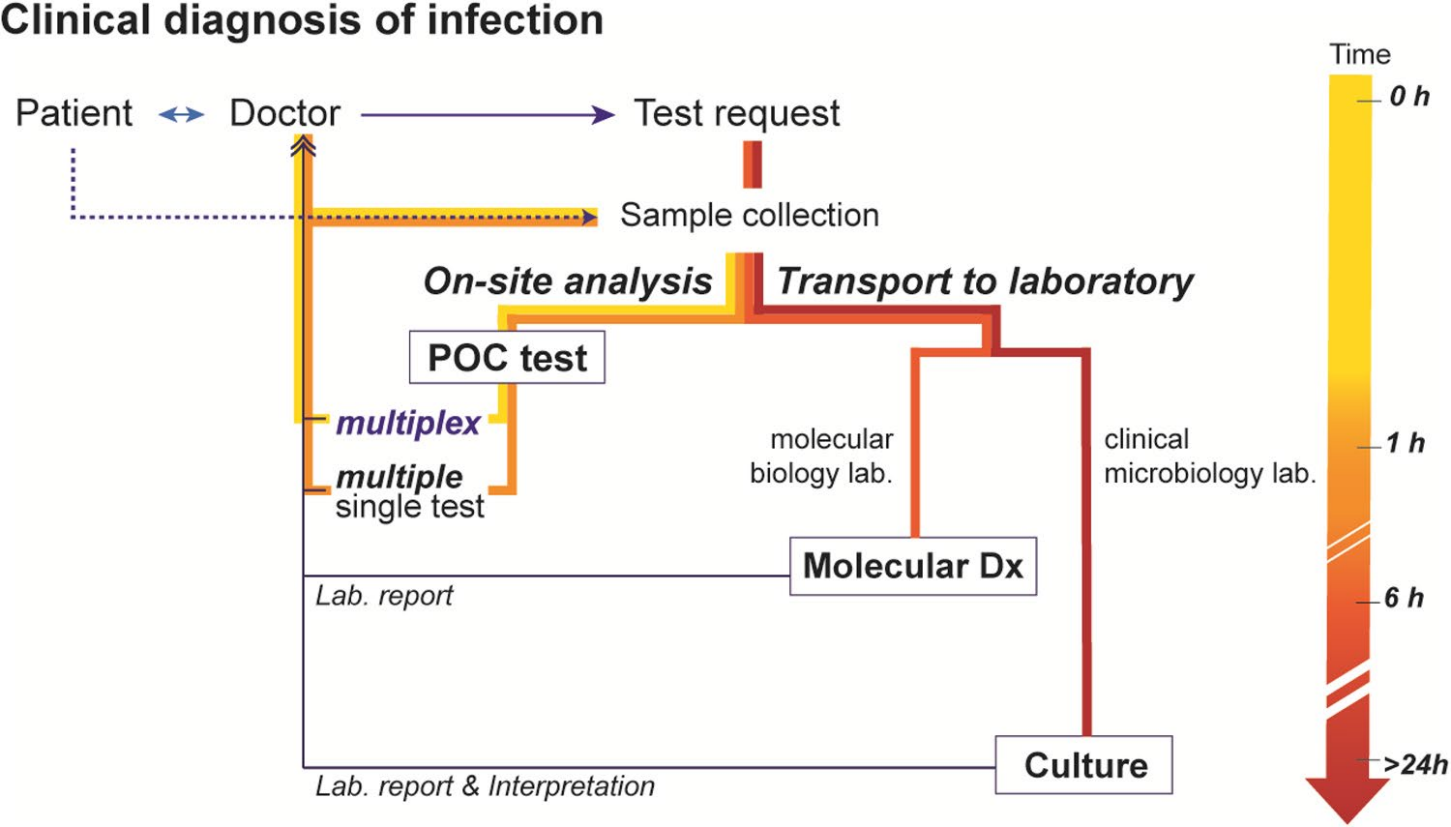
Clinical diagnosis of infection according to a diagnostic platform. The implementation of a multiplex point-of-care (POC) test reduces diagnostic cycles for faster and better treatment decisions

A personalized medicine and treatment regimen, which establishes clinical plans on a patient-by-patient basis in the treatment of infectious diseases, is a trending topic in the field of clinical microbiology and virology [1–5]. In general, patients with infectious illness present common symptoms. Symptoms including fever, cough, vomiting, abdominal pain, myalgia, and headache are often not sufficiently specific to differentiate the exact etiology for an infectious disease. In the paradigm of personalized medicine, the *sample in and answer out* approach of point-of-care (POC) diagnostics has the potential to empower physicians with the ability to make early evidence-based treatment decisions so that the right medication can be administered to the patient earlier, which can improve the prognosis. In fact, the treatment of infectious diseases are rarely considered to be model applications of personalized medicine; however, this perception is gradually changing due to a substantial increase in antimicrobial resistance (AMR) to antibiotics due to unnecessary usage [6–9]. Since experience-based empiric treatment is a possible driver of over-broad and unnecessary antibiotic usage, rapid POC diagnostic testing has become a promising solution to this problem. Molecular tests, such as polymerase chain reaction (PCR) and other nucleic acid-based amplification technologies (NAATs), have gradually replaced or augmented traditional laboratory techniques for pathogen identification in the form of POC testing, because these tests can detect fastidious or uncultivable microorganisms that indicate possible poly-microbial infection [10–12]. The successful migration of molecular diagnostics from universal laboratories to the clinical setting. could dramatically improve the accuracy and sensitivity of molecular POC testing (MDx POCT) [13–17]. In addition, it could lead to pathogen detection derived from a small amount of specimen, with delivery of results in just a few hours [13, 15, 17, 18]. Those rapid-throughput results would give providers confidence in prescribing the proper treatment (Fig. 1). Ultimately, exclusive molecular POC technologies may be implemented in the central care method in limited-resource settings.

Clinical microbiological and virological POC molecular diagnostics have initially addressed the optimization of technologies from centralized laboratories to the medical checkup field, regardless of the number of detectable pathogens [19]. The field has now expanded to encompass a comprehensive syndromic approach that simultaneously detects several similar pathogens. A few molecular POC tests exploit the early diagnosis of frequent infections, such as influenza [20] and healthcare-associated infections (HAIs) caused by *S. aureus*, *C. difficile*, enterococci or multi-resistant enterobacteria [21]. However, innovative multiplexed molecular POC testing has yet to be applied to infectious diseases because of the complex sample preparation required and the low throughput diagnosis. For instance, molecular diagnostics (MDx) involves numerous steps and reagents for sample preparation, including extractions and the purification of nucleic acids from specimens, amplification of a specific sequence, and confirmation of the target molecules [22]. Infectious disease MDx requires especially careful handling of specimens and test procedures to prevent the spread of viral molecules and aerosol from the sample and cross-contamination in the community [23, 24]. Infectious disease MDx POCT is also lacking in the number of pathogens that can be identified at once and has insufficient phenotypic information on pathogens. Fortunately, many studies have recently highlighted the significance of the multiplex capability of molecular POCT, because it provides rapid and high-accuracy answers that support quicker therapeutic decisions for the individual and at the public level [24]. Ultimately, multiplexed molecular POCT leads to pathogen-specific treatment and the use of narrow-spectrum antibiotics instead of excessive use of broad-spectrum antibiotics.

This review focuses on up-to-date progress in multiplexed molecular POCT for infectious diseases and efforts to overcome limitations. We have summarized information on recently approved devices and state-of-the-art developments through a comprehensive literature review. We also discuss the potential clinical impact of the recent POC approach and the possible hurdles that impede their practical application as a leading diagnostic test.

### Multiplex Molecular POC Testing: The Next Phase of Molecular Diagnostics

The implementation of NAATs has been a huge leap forward in test sensitivity and time-to-results. There are several molecular-based assays that meet the criteria for POC application and have been approved or are clinically available for the detection of single-key pathogens, such as influenza, *C. difficile,* or methicillin-resistant *Staphylococcus aureus* (MRSA). The first molecular test designed for POC was approved by the U.S. Food and Drug Administration (FDA) in 2015. ID NOW™ Influenza A & B 2, formerly known as Alere I influenza A & B 2, (Alere Scarborough Inc., Scarborough, ME, USA), provides highly sensitive results in 13 min or less. It is based on isothermal nucleic acid amplification technology, which uses nicking enzyme amplification reaction (NEAR) technology. As many emerging isothermal amplified POC applications have reached the proof-of-concept stage, ID NOW™ has successfully entered the infectious disease POC market for influenza A & B, Strep A and respiratory syncytial virus (RSV) testing [25–27]. ID NOW™, cobas^®^ Liat influenza A/B/RSV assays (Roche Molecular Systems, Pleasanton, CA, USA) and GeneXpert^®^ Xpress Flu/RSV (Cepheid, Sunnyvale, CA, USA) are known as Clinical Laboratory Improvement Amendments (CLIA)-waived (CW) assays that enable non-training personnel to obtain results within 30–50 min and are suitable for POCT [28]. However, ID NOW™ using NEAR technology is limited to the simultaneous detection and discrimination of multiple targets, which means that it includes individual assay panels for either influenza or RSV rather than a multiplexed test, whereas cobas® Liat influenza A/B/RSV assays and GeneXpert^®^ Xpress Flu/RSV are based on real-time reverse transcription-polymerase chain reaction (rtRT-PCR), which can detect both influenza A/B and RSV during a single run [29–31]. The binx *io* (binx Health Diagnostic, Boston, MA, USA), which combines PCR amplification and electrochemical detection technology, has recently received 510(k) clearance from the U.S. FDA for the dual targets of chlamydia (CT) and gonorrhea (NG) [32, 33]. These above-mentioned platforms have adopted single-use test units for sample preparation; nucleic acid extraction, purification and amplification, and the detection of biomarkers. While the laboratories have consistently demonstrated that the upgraded test kits have added target molecules, there are still significant challenges for the robust deployment of molecular POC systems; in particular, there is relatively low throughput in terms of simultaneous detection of copious pathogens. This limitation mainly arises from interference between multiple sets of primers and optical sources for target analysis. In detail, a large number of primers are required for simultaneous multi-pathogen detection, which results in preferential amplification between co-amplified genes and non-specific amplifications, such as primer dimer and mis-priming artifacts [34]. Moreover, the optics of current analytical instruments and sequencing probes (e.g., fluorescence label) bring a limited number of targets to deflect from an overlapping wavelength in a single reaction and laser source [35]. The novel design of probe formats and combination of microfluidic technologies has been proposed to be the solution for improved simultaneous detection of a larger number of targets. For instance, FilmArray^®^ (BioFire, Salt Lake City, UT, USA) exemplifies this idea of a technical breakthrough enabling simultaneous detection of multiple targets. The microfluidic pouch system connects to a module for DNA extraction and purification and employs a post-PCR melting curve analysis that differentiates targets based on the distinct melting temperature (*T*_m_) peaks for each target. This allows for the multiplexed detection of multiple targets. Another example is the FDA-cleared Verigene (Nanosphere, Northbrook, IL, USA) system, which that uses gold nanoparticle probes and a microarray for the detection of bacterial pathogens and several resistance markers. However, neither platform is classified as a POC test.

### Recent Multiplex Molecular POC Testing for the Broad Detection of pathogens: The Way Forward

**Fig. 2:**
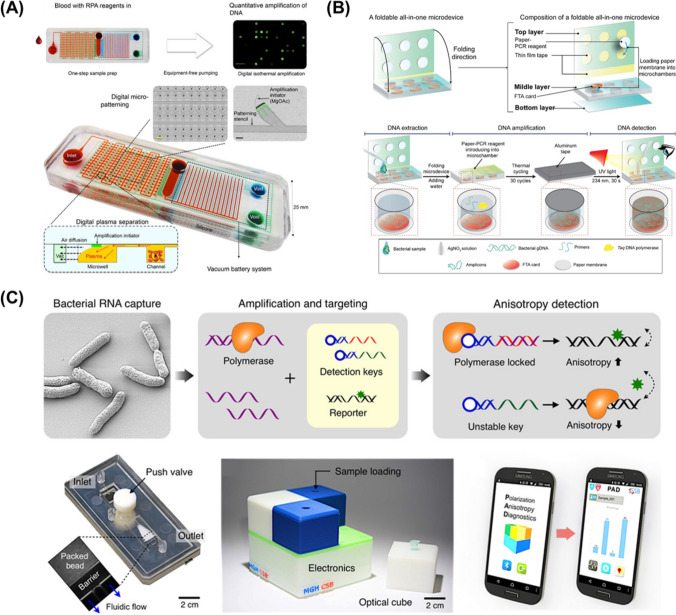
Integrated molecular diagnostic assay-based microfluidic devices. **a** SIMPLE chip for blood sample prep, simultaneous digital amplification, and quantitative nucleic acid testing with minimal handling and no-power source. **b** A foldable all-in-one microdevice for the entire process of multiplex pathogen molecular test by a folding and stacking motion. **c** Polarized anisotropy diagnostics (PAD) system for the detection of bacterial total RNA. (**a** From Lee, L.P. et al., Sci Adv 2017, 3 (3), e1501645 under open access license CC BY-NC, reprinted with permission from AAAS/**b** Reprinted from Lee, N. Y. et al., Sens. Actuators B Chem 2020, 314, 128,057. Copyright by Elsevier/ (C) From Lee, H. et al., Sci Adv 2016, 2 (5), e1600300 under open access license CC BY-NC, Reprinted with permission AAAS)

Microfluidic devices for molecular diagnostics have been less commercialized over the last few years compared to the immense academic interest focusing on the miniaturization of molecular biological techniques, including target gene amplification onto microfluidic devices. However, there is an increasing need to integrate microfluidic devices with other applications, such as sample pre-treatment, fluid control, and multiplex capability (Fig. 2). For example, Liu et al. reported a microfluidic cassette that is able to capture, concentrate, amplify, and optically detect the Zika virus (ZIKV) in a single sample-to-answer chip [36]. They have established a silica-based nucleic acid isolation membrane in a microchannel and thermally insulated a portable heating cup for PCR reactions in an emergency. Even though the POC molecular test is composed of a modest set with a chip and heater, the obtained result was reasonable, with a high sensitivity of 5 PFU ZIKV per sample within 40 min. In accordance with the emergence of nucleic acid amplification technologies, multiplex microfluidics have also been developed with the goal of eliminating the heating cycle apparatus. Lee et al. demonstrated a self-powered integrated microfluidic POC low-cost enabling (SIMPLE) chip [37]. It contains an isothermal recombinase polymerase amplification initiator patterning on the well of the channel, autonomous plasma separation into 224 microwells for digital nucleic acid amplification, and a vacuum battery on the chip. The integrated options have been developed as a lab-on-a-chip for molecular testing that automatically prepares plasma from whole blood into hundreds of microwells, directly amplifies the independent target in every well due to micropatterning of the initiator, and can run without a power source or external pumps. The SIMPLE chip also detects *Staphylococcus aureus* DNA from human blood samples in 30 min with separation, amplification, and digital quantitative nucleic acid readouts. The result simplifies molecular testing, making it portable, and available for rapid medical decisionmaking. On the other hand, NAATs that utilize paper substrate matrices provide a low-cost, easy-to-store, and portable platform. Klapperich’s et al. developed a single paper-fluidic-chip-based lateral flow assay, which allows for the completion of NAAT steps from sample to result [38]. The device drives the isolation of DNA with reagents on the chip, followed by isothermal loop-mediated amplification and a pump-free immunochromatographic assay. All processes are operated on paper, which passively move fluids through wicking, capillary force, and sample handling. The authors have detected 16 subtypes of human papillomavirus (HPV) DNA directly from cervical specimens in less than 1 h with an integrated on-chip assay that produces an immediate visual readout.

Numerous studies have highlighted the importance of a syndromic approach in a multiplex molecular diagnostic test [39–41]. A syndromic approach in molecular diagnosis achieves the simultaneous detection of genetic material from pathogens of different species and even different taxonomic levels in a single test. For a comprehensive group of pathogens that could cause a specific syndrome, such as food poisoning or an upper respiratory infection, it may help effectively manage outbreaks with seasonal pathogen screening and detect community- and healthcare-associated outbreaks [42, 43]. Lee et al. presented a foldable all-in-one microdevice for simultaneous detection of three foodborne pathogens, *Salmonella spp., Staphylococcus aureus, and Escherichia coli* O157:H7, and a multi-drug-resistant bacteria *Acinetobacter baumannii* [44]. The platform is feasible for accomplishing PCR preparation, amplification, and multiplex optical detection of pathogens in less than 2 h. The thin polymethyl methacrylate (PMMA)-molded microdevice consists of PCR reagent pre-stored filter paper disks and Flinders Technology Associates cards for cell lysis, protein denaturation, and DNA extraction. Consequently, adding silver nitrate results in the multiplex pathogen detection system for direct colorimetric detection without an expert analytical tool. To quantify the bacterial cell, Mu et al. reported an integrated multiplex digital recombinase polymerase amplification (imdRPA) microfluidic chip, which enables to detect $1\times {10}^{1}$ foodborne bacterial cells of each species within 45 min [45]. They adopted a magnetic bead-based DNA extraction component with a digital RPA region including 12,800 chambers for simultaneous detection of the targets and control, and then quantitatively analyzed the multiplex foodborne pathogen from the fluorescence signal of the DNA probe. These microdevices are highly suitable for the detection of numerous pathogens by increasing the number of channels in a chip for a syndromic diagnosis approach. Among various emerging breakthrough technologies, Park et al. validated a new detection system based on polarization anisotropy diagnostics (PAD), which measures changes in fluorescence anisotropy when detection probes recognize the nucleic acid of the target bacteria [46]. This assay supports a universal capture key that targets a conserved region of 16S rRNA in different bacterial species. PAD was applied on-site in clinical HAI diagnostics and achieved an accuracy comparable to that of bacterial culture, and with a shorter turnaround time (~ 2 h). One of the commercial molecular POCs, an STD Direct Flow Chip Kit (Master Diagnóstica, Granada, Spain), was evaluated for the detection of up to nine different pathogen species of sexually transmitted diseases (STDs) from clinical specimens in a single reaction 47. The kit achieves molecular diagnosis based on a multiplex PCR and automatic hybridization onto a microarray with specific oligo probes and broad clinical specimens while avoiding DNA purification steps. From a total of 633 specimens, the direct PCR-analysis results were 98.4% and 99.9% for sensitivity and specificity, respectively.

Numerous studies involving multiplex molecular POC are now moving toward extensive diagnosis capability, but few kits have been approved for the detection of multiple pathogens based on clinical syndromes (Table 1). For example, the GenePOC technology platform (Meridian Bioscience, Inc., Quebec, Canada) integrates a microfluidic centripetal device to provide molecular diagnostic technologies in POC [48, 49]. Within 1 h and with less than 1 min of hands-on time, the GenePOC platform enables fully automated nucleic acid-based testing for infectious microorganisms. Overall, it can process a wide range of clinical samples with up to 12 targets. Culture-independent direct pathogen detection approaches are included in most advanced technologies, such as Abbott’s IRIDICA and T2 Biosystems’ magnetic resonance technology, which are capable of detecting many microorganisms. Abbott’s IRIDICA combines a set of broad-range PCRs and electrospray ionization-mass spectrometry (PCR/ESI–MS). Herein, the PCRs amplify gene encoding 16S ribosomal RNA and detect the housekeeping gene region. Otherwise, T2 relies on changes in a sample’s T2 magnetic resonance (T2MR^®^) signal, which is caused by hybridization of the PCR-amplified pathogen DNA and the capture of probe-decorated nanoparticles [50]. The T2 system requires 1 mL uncultured whole blood and provides results in approximately 3 h, with a claimed limit of detection as low as 1 colony-forming unit (CFU)/ml. However, this assay has currently been confirmed to detect only the five most common *Candida* species (which account for 95% of candidemia) but is able to provide antifungal susceptibility data [51]. Due to growing demand for multiplex molecular POC testing, major companies in the in vitro diagnostics (IVD) market are focused on pipeline expansion and fortifying their partnerships. For instance, the molecular diagnostic company Qiagen (Hilden, Germany) has agreed to acquire Stat-Dx (Barcelona, Spain), which has developed a fully integrated one-step molecular test for common syndromes. QIAstat-Dx (formerly Stat-Dx DiagCORE^®^) is a multiplex molecular diagnostic system that enables fast, cost-effective, and flexible syndromic testing. The system has received CE-IVD certification for a respiratory panel that detects 21 pathogens and will be able to process up to 48 molecular targets at once, which will permit the diagnosis of serious respiratory and gastrointestinal infections.

**Table 1:** Analytical spectrum of molecular near-patient and point-of-care testing for infectious diseases

System	Multiplexity	Virology	Microbiology
ID NOW™ Abbott	Single	FLU A/B, RSV, SARS-CoV-2^a^	GAS
cobas^®^ Liat^®^ Roche	Single	FLU A/B	GAS, CDI
	Multiple	FLU A/B + RSV	
GeneXpert Omni Cepheid	Single	EBO, EV, FLU A/B, HPV, HBV, HCV, HIV, SARS-CoV-2*	GAS, TV, CT, GBS, CDI, CARBA-R, MRSA, NORO, VRE
	Multiple	FLU A/B + RSV	MTB + RIF, CT + NG, MRSA + SA
binx *io* binx health, inc	Single	HPV, HIV, HCV, HBV, HSV-2	CT, NG, TV, TP, MG
	Multiple		CT + NG, CT + NG + TV, CT + NG + TV + MG
FilmArray^b^ BioMerieux	Multiple	Respiratory Panel: 18 viruses, 4 bacteria	
		Blood Culture Identification 2 (BCID2) Panel: 26 bacteria, 7 yeast, 10 antibiotic resistance genes	
		Gastrointestinal (GI) Panel: 5 viruses, 7 bacteria, 6 Diarrheagenic E. coli/Shigella, 4 parasites	
		Meningitis-Encephalitis (ME) Panel: 7 viruses, 6 bacteria, 1 yeast	
		Pneumonia *plus* Panel: 18 bacteria, 7 antibiotic resistance genes, 9 viruses	
Verigene^b^ Nanosphere	Single		CDI
	Multiple	Enteric Pathogens Test: 2 viruses, 6 bacteria, 2 toxins	
		Respiratory Pathogens *Flex* test: 13 viruses, 2 bacteria	
		Blood Culture Nucleic Acid Testing Panels: 1. Gram-positive: 8 species, 1 group, 4 genus, 3 resistance markers 2. Gram-negative: 5 species, 4 genus, 6 resistance markers	
GenePOC Meridian Bioscience, inc	Single		CDI, GBS
Q-POC QuantuMDx	Single	HPV, MDR-TB	
	Multiple		CT + NG
QIAstat-Dx Qiagen	Multiple	Respiratory Panel: 18 viral, 3 bacterial pathogens	
		Respiratory SARS-CoV-2 panel*: 19 viral pathogens including SARS-CoV-2, 3 bacterial pathogens	
		Gastrointestinal Panel: 6 viral, 14 bacterial, 4 parasitic pathogens	
T2MR^®^ T2 Biosystems	Single		Candida
	Multiple		ESKAPE

*FLU A/B* Influenza A/B, *RSV* respiratory syncytial virus, *SARS-CoV-2* severe acute respiratory syndrome coronavirus 2, *GAS* group A streptococci, *CDI* *Clostridium difficile*, *EBO* Ebola virus, *EV* enterovirus, *HPV* human papillomavirus, *HBV* Hepatitis B virus, *HCV* Hepatitis C virus, *HIV* Human immunodeficiency virus, *TV* *Trichomonas vaginalis*, *CT* *Chlamydia trachomatis*, *GBS* group B streptococci, *CARBA-R* carbapenem-resistant Enterobacteriaceae, *MRSA* methicillin-resistant *Staphylococcus aureous*, *NORO* Norovirus, *VRE* Vancomycin-resistant enterococci, *MTB* *Mycobacterium tuberculosis*, *RIF* Rifampicin resistance, *NG* *Neisseria gonorrhoeae*, *SA* *Staphylococcus*, *HSV-2* Herpes simplex virus type two, *TP* *Treponema pallidum*, *MG* *Mycoplasma genitalium*, *MDR-TB* Multi-drug resistance tuberculosis, *ESKAPE* *Enterococcus faecium/Staphylococcus aureus/Klebsiella pneumoniae/Pseudomonas aeruginosa/Escherichia coli*

^a^For use under an Emergency-Use Authorization (EUA, 2020.09.24)

^b^Not POC

### Persistent Challenges

A few years ago, molecular POC diagnosis was limited to testing only the most common pathogens. This lack in diagnostics capability led to further downstream tests or unnecessary prescription of antibiotics. The recently commercialized multiplex molecular assays enable simultaneous detection and identification of multiple pathogens associated with clinical syndromes with regard to bloodstream, respiratory, gastrointestinal (GI), or central nervous system infections [40]. As the demand for syndromic testing in molecular diagnostics increases, there is an ongoing debate about whether these tests will be applied as front-line diagnostics techniques for all patients or will be limited to specific patients and regions [39, 52]. Even if multiplex test menus, i.e., combinations of pathogens, are properly linked to confirm the clinical syndromes, it may be ineffective cost-wise to test all specimens. Thus, syndromic testing must be customizable and personalized for patient-specific needs to exclude over-testing and excess patient medical bills. Consequently, an optimized technical model should be applied in the development of POC tests using a syndromic approach. In addition, incorporating multiplex molecular POC tests in clinical practice may encourage a change in the workflow of existing central laboratory tests. Currently, it is important to consider whether multiplex MDx POCT would replace or be conducted in addition to existing tests. For instance, many studies have applied ultrafast thermocycling approaches that perform efficient PCR processes through rapid temperature control and a high transfer rate of heating and cooling, resulting in a small reaction volume and photophysical effect [11, 53, 54]. The developed factors consist of miniaturized thermocyclers for ultrafast PCR, including capillary tubes with air heating, a microfluidic device, and a photonic microwave-based resistor-mediated or convective thermocycler [55–59]. In fact, the key prerequisite is that performance should be equal in terms of sensitivity and specificity when it is compared to tests that are conducted in a central laboratory. Another consideration is that it should be available to use at the primary care level, even in resource-limited settings. Although the sensitivity and specificity are similar to the gold standard method, performance should be achieved equally, even in a resource-constrained setting. When a comprehensive test such as the multiplex MDx POC test is actualized to administer medical checkup performance, it has the potential to become a dominant diagnostic test.

## Conclusion

Evolving POC molecular diagnostics are closer to realization in the medical field. The successful migration of a molecular POC test has changed the trends of centralized laboratory testing from general molecular diagnostics with highly skilled experts and complex equipment to the more comprehensive molecular diagnostics in a clinical setting using simpler instruments. The emergence of a new CLIA-waived molecular POC platform has received major attention in the global in vitro diagnostics (IVD) market, because traditional POC tests, *e.g.,* lateral flow, are commonly known to lack sensitivity. Those established tests could be replaced by rapid molecular diagnostics that are simple, easy to use, and highly accurate compared to the former gold standard methods. In this review, we focused on recent progress in molecular POC testing for infectious diseases and summarize the available information regarding recently approved devices and state-of-the-art developments. The implementation of multiplex molecular POC testing will result in unprecedented changes in antimicrobial therapeutic decisions, management, and control of infectious diseases. Despite technological advances, there are still a great deal of challenges for multiplex molecular POC tests in clinical practice, such as moderate test performance, complexity that make it difficult to use in many clinical settings, limited access to testing, and high costs. Considering that countless researchers have encouraged finding a way to overcome these challenges, we believe that multiplex molecular POC will become a powerful principal diagnostic test.
